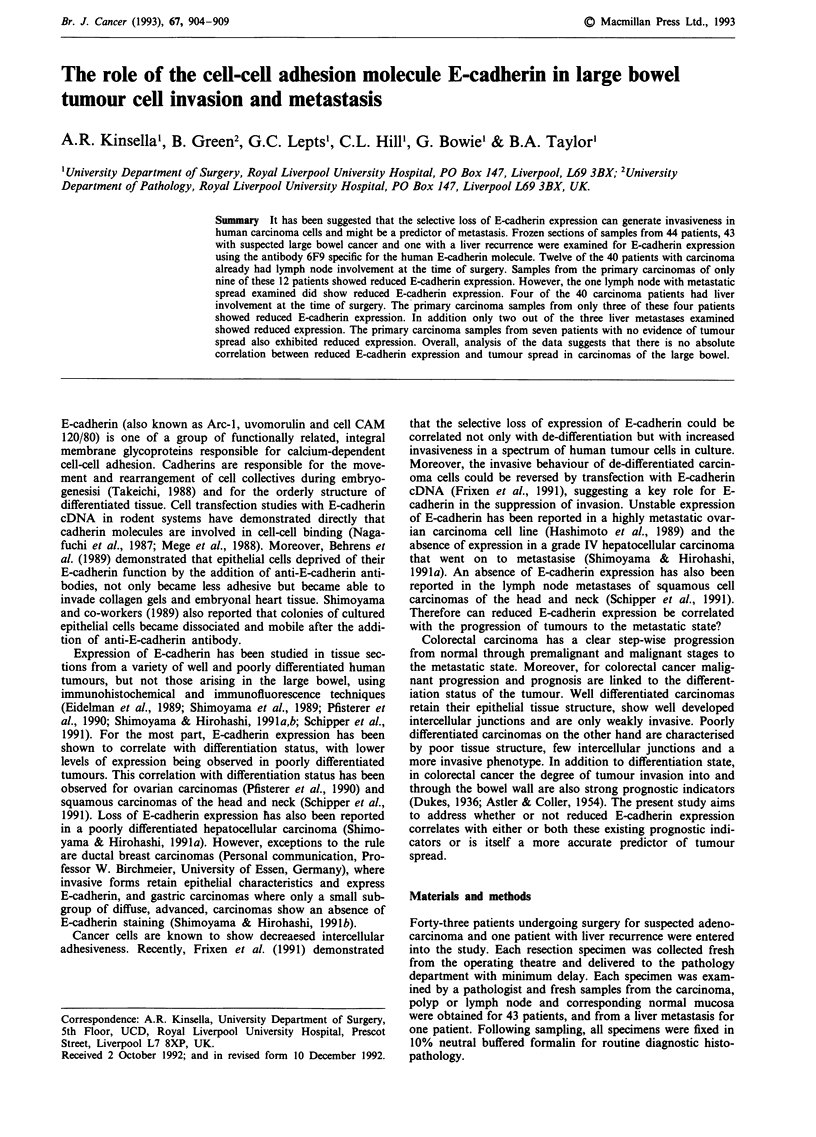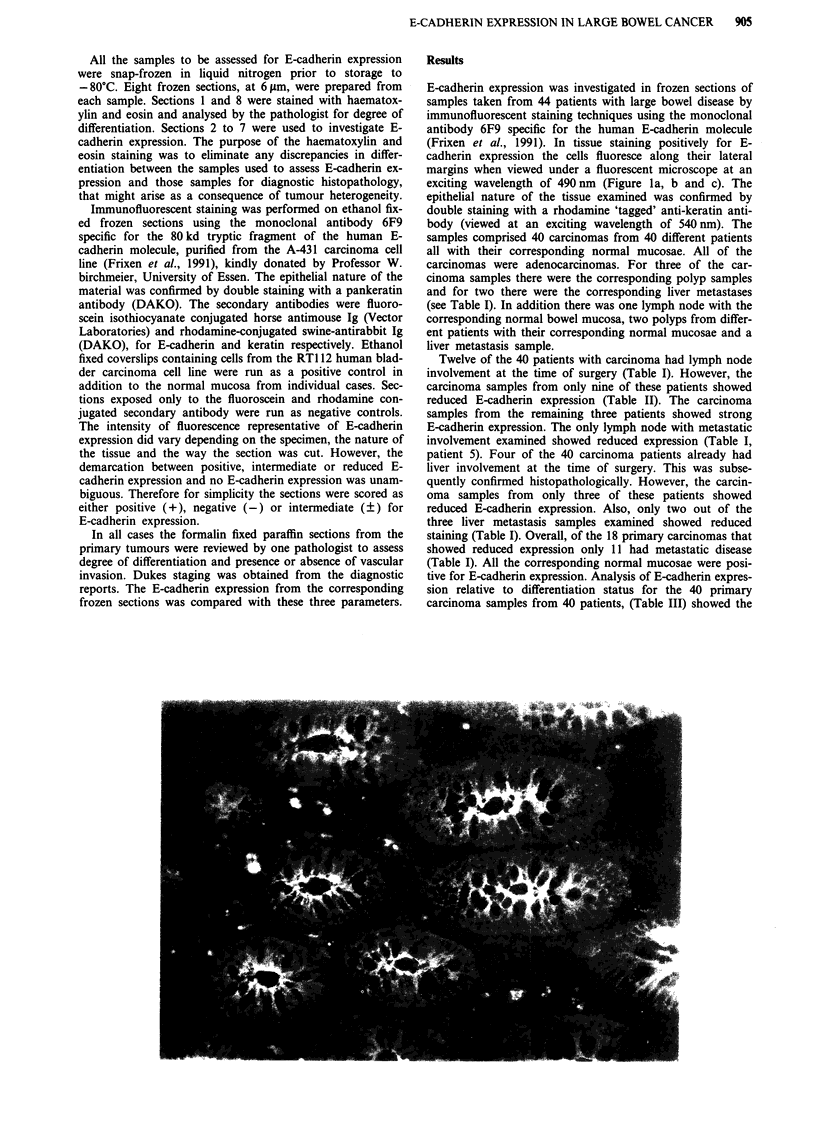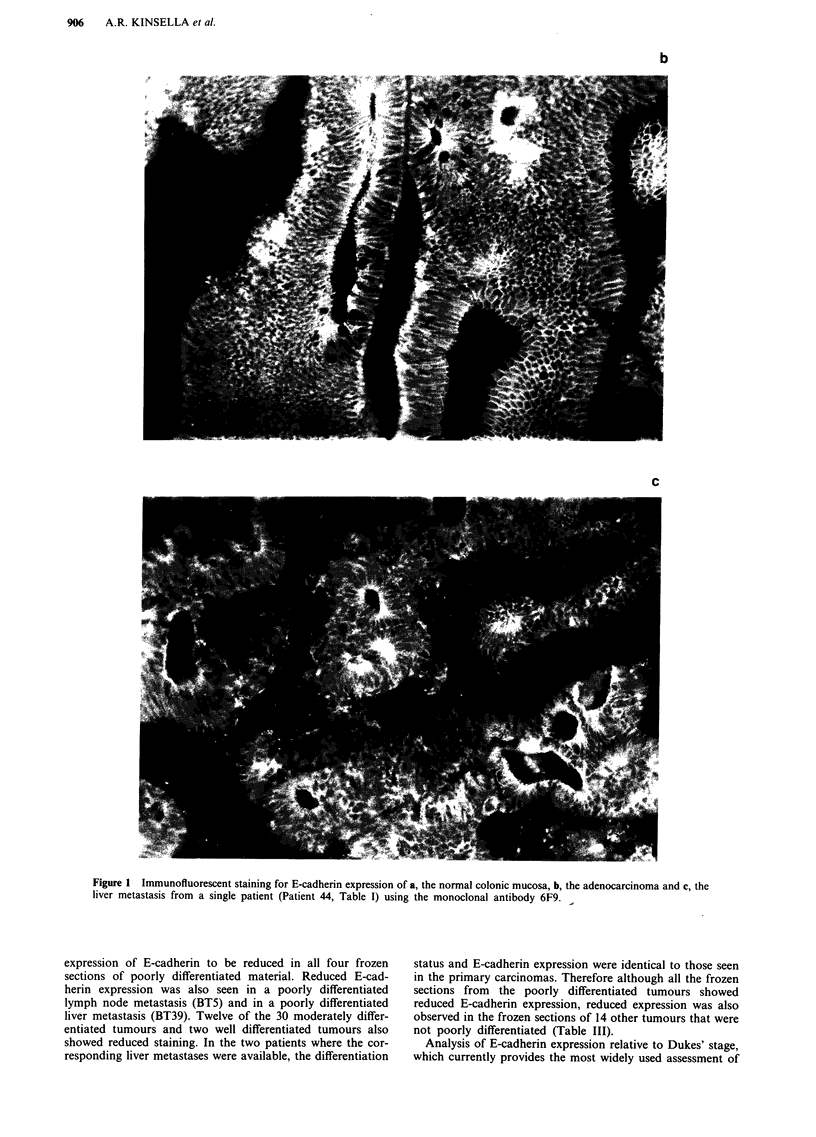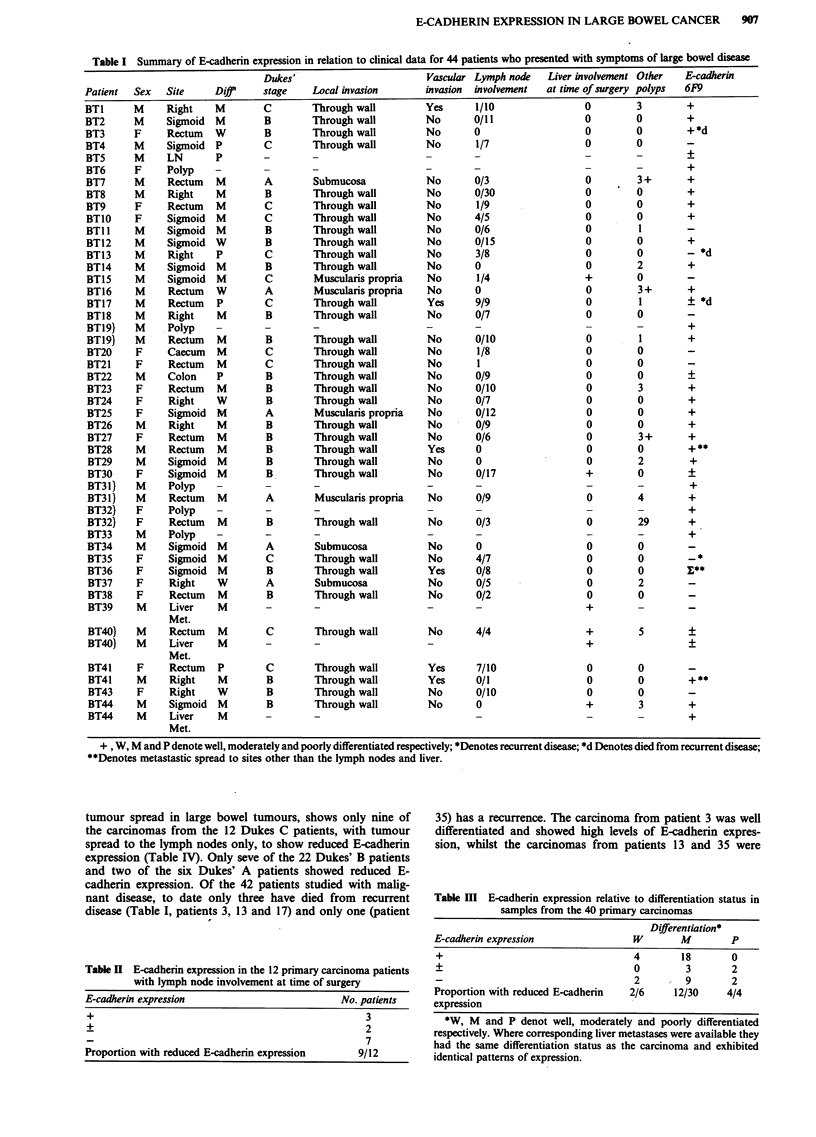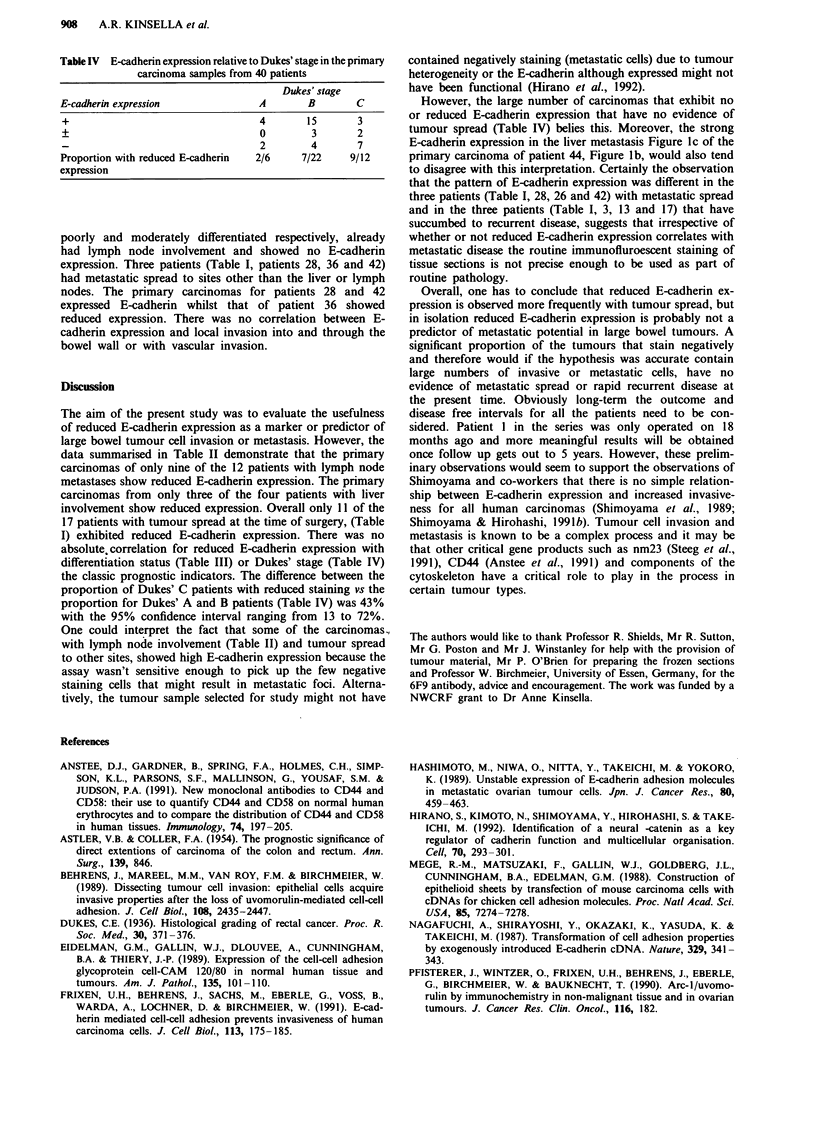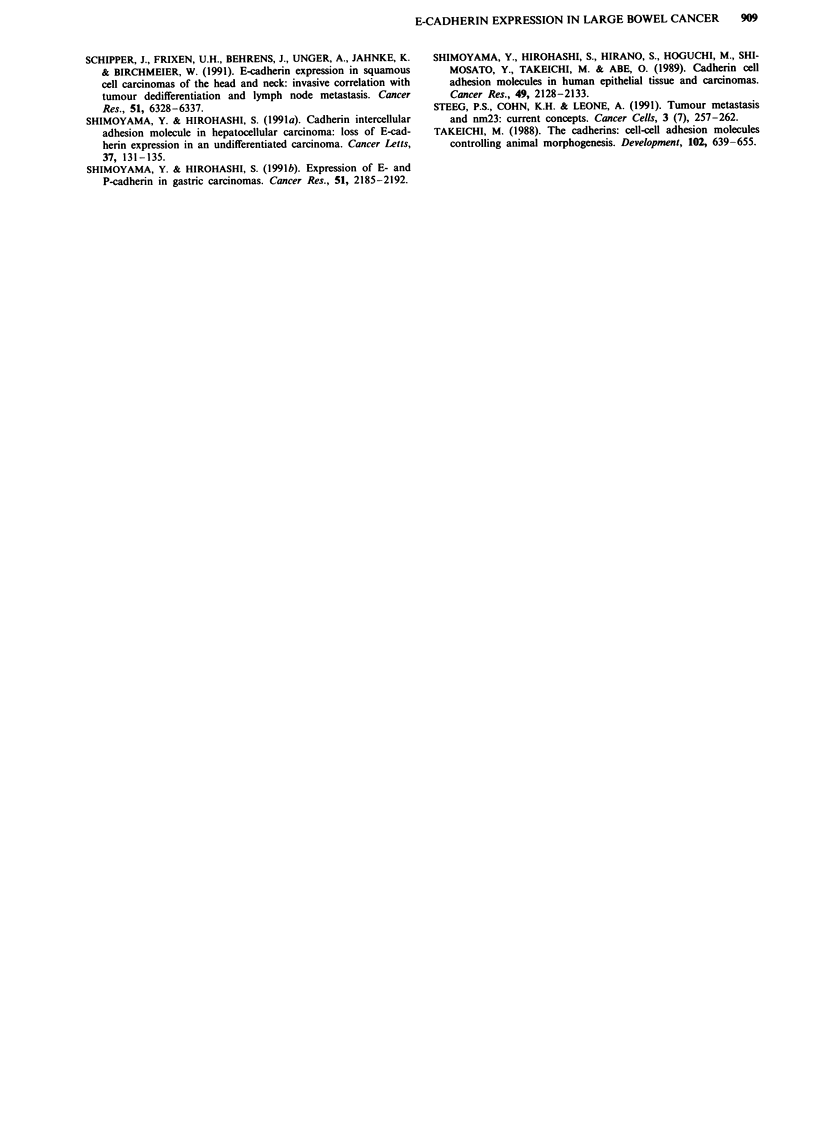# The role of the cell-cell adhesion molecule E-cadherin in large bowel tumour cell invasion and metastasis.

**DOI:** 10.1038/bjc.1993.169

**Published:** 1993-05

**Authors:** A. R. Kinsella, B. Green, G. C. Lepts, C. L. Hill, G. Bowie, B. A. Taylor

**Affiliations:** University Department of Surgery, Royal Liverpool University Hospital, UK.

## Abstract

**Images:**


Br. J. Cancer (1993), 67, 904-909 © Macmillan Press Ltd., 1993

### The role of the cell-cell adhesion molecule E-cadherin in large bowel tumour cell invasion and metastasis

A.R. Kinsella[1], B. Green[2], G.C. Lepts[1], C.L. Hill[1], G. Bowie[1] & B.A. Taylor[1]

[1]*University Department of Surgery, Royal Liverpool University Hospital, PO Box 147, Liverpool, L69 3BX;* [2]*University Department of Pathology, Royal Liverpool University Hospital, PO Box 147, Liverpool L69 3BX, UK.*

**Summary** It has been suggested that the selective loss of E-cadherin expression can generate invasiveness in human carcinoma cells and might be a predictor of metastasis. Frozen sections of samples from 44 patients, 43 with suspected large bowel cancer and one with a liver recurrence were examined for E-cadherin expression using the antibody 6F9 specific for the human E-cadherin molecule. Twelve of the 40 patients with carcinoma already had lymph node involvement at the time of surgery. Samples from the primary carcinomas of only nine of these 12 patients showed reduced E-cadherin expression. However, the one lymph node with metastatic spread examined did show reduced E-cadherin expression. Four of the 40 carcinoma patients had liver involvement at the time of surgery. The primary carcinoma samples from only three of these four patients showed reduced E-cadherin expression. In addition only two out of the three liver metastases examined showed reduced expression. The primary carcinoma samples from seven patients with no evidence of tumour spread also exhibited reduced expression. Overall, analysis of the data suggests that there is no absolute correlation between reduced E-cadherin expression and tumour spread in carcinomas of the large bowel.

E-cadherin (also known as Arc-1, uvomorulin and cell CAM 120/80) is one of a group of functionally related, integral membrane glycoproteins responsible for calcium-dependent cell-cell adhesion. Cadherins are responsible for the movement and rearrangement of cell collectives during embryogenesisi (Takeichi, 1988) and for the orderly structure of differentiated tissue. Cell transfection studies with E-cadherin cDNA in rodent systems have demonstrated directly that cadherin molecules are involved in cell-cell binding (Nagafuchi *et al.*, 1987; Mege *et al.*, 1988). Moreover, Behrens *et al.* (1989) demonstrated that epithelial cells deprived of their E-cadherin function by the addition of anti-E-cadherin antibodies, not only became less adhesive but became able to invade collagen gels and embryonal heart tissue. Shimoyama and co-workers (1989) also reported that colonies of cultured epithelial cells became dissociated and mobile after the addition of anti-E-cadherin antibody.

Expression of E-cadherin has been studied in tissue sections from a variety of well and poorly differentiated human tumours, but not those arising in the large bowel, using immunohistochemical and immunofluorescence techniques (Eidelman *et al.*, 1989; Shimoyama *et al.*, 1989; Pfisterer *et al.*, 1990; Shimoyama & Hirohashi, 1991*a,b*; Schipper *et al.*, 1991). For the most part, E-cadherin expression has been shown to correlate with differentiation status, with lower levels of expression being observed in poorly differentiated tumours. This correlation with differentiation status has been observed for ovarian carcinomas (Pfisterer *et al.*, 1990) and squamous carcinomas of the head and neck (Schipper *et al.*, 1991). Loss of E-cadherin expression has also been reported in a poorly differentiated hepatocellular carcinoma (Shimoyama & Hirohashi, 1991*a*). However, exceptions to the rule are ductal breast carcinomas (Personal communication, Professor W. Birchmeier, University of Essen, Germany), where invasive forms retain epithelial characteristics and express E-cadherin, and gastric carcinomas where only a small subgroup of diffuse, advanced, carcinomas show an absence of E-cadherin staining (Shimoyama & Hirohashi, 1991*b*).

Cancer cells are known to show decreaesed intercellular adhesiveness. Recently, Frixen *et al.* (1991) demonstrated that the selective loss of expression of E-cadherin could be correlated not only with de-differentiation but with increased invasiveness in a spectrum of human tumour cells in culture. Moreover, the invasive behaviour of de-differentiated carcinoma cells could be reversed by transfection with E-cadherin cDNA (Frixen *et al.*, 1991), suggesting a key role for E-cadherin in the suppression of invasion. Unstable expression of E-cadherin has been reported in a highly metastatic ovarian carcinoma cell line (Hashimoto *et al.*, 1989) and the absence of expression in a grade IV hepatocellular carcinoma that went on to metastasise (Shimoyama & Hirohashi, 1991*a*). An absence of E-cadherin expression has also been reported in the lymph node metastases of squamous cell carcinomas of the head and neck (Schipper *et al.*, 1991). Therefore can reduced E-cadherin expression be correlated with the progression of tumours to the metastatic state?

Colorectal carcinoma has a clear step-wise progression from normal through premalignant and malignant stages to the metastatic state. Moreover, for colorectal cancer malignant progression and prognosis are linked to the differentiation status of the tumour. Well differentiated carcinomas retain their epithelial tissue structure, show well developed intercellular junctions and are only weakly invasive. Poorly differentiated carcinomas on the other hand are characterised by poor tissue structure, few intercellular junctions and a more invasive phenotype. In addition to differentiation state, in colorectal cancer the degree of tumour invasion into and through the bowel wall are also strong prognostic indicators (Dukes, 1936; Astler & Coller, 1954). The present study aims to address whether or not reduced E-cadherin expression correlates with either or both these existing prognostic indicators or is itself a more accurate predictor of tumour spread.

### Materials and methods

Forty-three patients undergoing surgery for suspected adenocarcinoma and one patient with liver recurrence were entered into the study. Each resection specimen was collected fresh from the operating theatre and delivered to the pathology department with minimum delay. Each specimen was examined by a pathologist and fresh samples from the carcinoma, polyp or lymph node and corresponding normal mucosa were obtained for 43 patients, and from a liver metastasis for one patient. Following sampling, all specimens were fixed in 10% neutral buffered formalin for routine diagnostic histopathology.

Correspondence: A.R. Kinsella, University Department of Surgery, 5th Floor, UCD, Royal Liverpool University Hospital, Prescot Street, Liverpool L7 8XP, UK.
Received 2 October 1992; and in revised form 10 December 1992.

All the samples to be assessed for E-cadherin expression were snap-frozen in liquid nitrogen prior to storage to −80°C. Eight frozen sections, at 6 μm, were prepared from each sample. Sections 1 and 8 were stained with haematoxylin and eosin and analysed by the pathologist for degree of differentiation. Sections 2 to 7 were used to investigate E-cadherin expression. The purpose of the haematoxylin and eosin staining was to eliminate any discrepancies in differentiation between the samples used to assess E-cadherin expression and those samples for diagnostic histopathology, that might arise as a consequence of tumour heterogeneity.

Immunofluorescent staining was performed on ethanol fixed frozen sections using the monoclonal antibody 6F9 specific for the 80 kd tryptic fragment of the human E-cadherin molecule, purified from the A-431 carcinoma cell line (Frixen *et al.*, 1991), kindly donated by Professor W. birchmeier, University of Essen. The epithelial nature of the material was confirmed by double staining with a pankeratin antibody (DAKO). The secondary antibodies were fluoroscein isothiocyanate conjugated horse antimouse Ig (Vector Laboratories) and rhodamine-conjugated swine-antirabbit Ig (DAKO), for E-cadherin and keratin respectively. Ethanol fixed coverslips containing cells from the RT112 human bladder carcinoma cell line were run as a positive control in addition to the normal mucosa from individual cases. Sections exposed only to the fluoroscein and rhodamine conjugated secondary antibody were run as negative controls. The intensity of fluorescence representative of E-cadherin expression did vary depending on the specimen, the nature of the tissue and the way the section was cut. However, the demarcation between positive, intermediate or reduced E-cadherin expression and no E-cadherin expression was unambiguous. Therefore for simplicity the sections were scored as either positive (+), negative (−) or intermediate (±) for E-cadherin expression.

In all cases the formalin fixed paraffin sections from the primary tumours were reviewed by one pathologist to assess degree of differentiation and presence or absence of vascular invasion. Dukes staging was obtained from the diagnostic reports. The E-cadherin expression from the corresponding frozen sections was compared with these three parameters.

### Results

E-cadherin expression was investigated in frozen sections of samples taken from 44 patients with large bowel disease by immunofluorescent staining techniques using the monoclonal antibody 6F9 specific for the human E-cadherin molecule (Frixen *et al.*, 1991). In tissue staining positively for E-cadherin expression the cells fluoresce along their lateral margins when viewed under a fluorescent microscope at an exciting wavelength of 490 nm (Figure 1a, b and c). The epithelial nature of the tissue examined was confirmed by double staining with a rhodamine 'tagged' anti-keratin antibody (viewed at an exciting wavelength of 540 nm). The samples comprised 40 carcinomas from 40 different patients all with their corresponding normal mucosae. All of the carcinomas were adenocarcinomas. For three of the carcinoma samples there were the corresponding polyp samples and for two there were the corresponding liver metastases (see Table I). In addition there was one lymph node with the corresponding normal bowel mucosa, two polyps from different patients with their corresponding normal mucosae and a liver metastasis sample.

Twelve of the 40 patients with carcinoma had lymph node involvement at the time of surgery (Table I). However, the carcinoma samples from only nine of these patients showed reduced E-cadherin expression (Table II). The carcinoma samples from the remaining three patients showed strong E-cadherin expression. The only lymph node with metastatic involvement examined showed reduced expression (Table I, patient 5). Four of the 40 carcinoma patients already had liver involvement at the time of surgery. This was subsequently confirmed histopathologically. However, the carcinoma samples from only three of these patients showed reduced E-cadherin expression. Also, only two out of the three liver metastasis samples examined showed reduced staining (Table I). Overall, of the 18 primary carcinomas that showed reduced expression only 11 had metastatic disease (Table I). All the corresponding normal mucosae were positive for E-cadherin expression. Analysis of E-cadherin expression relative to differentiation status for the 40 primary carcinoma samples from 40 patients, (Table III) showed the a

**Figure 1** Immunofluorescent staining for E-cadherin expression of **a**, the normal colonic mucosa, **b**, the adenocarcinoma and **c**, the liver metastasis from a single patient (Patient 44, Table I) using the monoclonal antibody 6F9.

expression of E-cadherin to be reduced in all four frozen sections of poorly differentiated material. Reduced E-cadherin expression was also seen in a poorly differentiated lymph node metastasis (BT5) and in a poorly differentiated liver metastasis (BT39). Twelve of the 30 moderately differentiated tumours and two well differentiated tumours also showed reduced staining. In the two patients where the corresponding liver metastases were available, the differentiation status and E-cadherin expression were identical to those seen in the primary carcinomas. Therefore although all the frozen sections from the poorly differentiated tumours showed reduced E-cadherin expression, reduced expression was also observed in the frozen sections of 14 other tumours that were not poorly differentiated (Table III).

Analysis of E-cadherin expression relative to Dukes' stage, which currently provides the most widely used assessment of

**Table I** Summary of E-cadherin expression in relation to clinical data for 44 patients who presented with symptoms of large bowel disease

| *Patient* | *Sex* | *Site* | *Diff*$^n$ | *Dukes' stage* | *Local invasion* | *Vascular invasion* | *Lymph node involvement* | *Liver involvement at time of surgery* | *Other polyps* | *E-cadherin 6F9* |
|---|---|---|---|---|---|---|---|---|---|---|
| BT1 | M | Right | M | C | Through wall | Yes | 1/10 | 0 | 3 | + |
| BT2 | M | Sigmoid | M | B | Through wall | No | 0/11 | 0 | 0 | + |
| BT3 | F | Rectum | W | B | Through wall | No | 0 | 0 | 0 | +*d |
| BT4 | M | Sigmoid | P | C | Through wall | No | 1/7 | 0 | 0 | – |
| BT5 | M | LN | P | – | – | – | – | – | – | ± |
| BT6 | F | Polyp | – | – | – | – | – | – | – | + |
| BT7 | M | Rectum | M | A | Submucosa | No | 0/3 | 0 | 3+ | + |
| BT8 | M | Right | M | B | Through wall | No | 0/30 | 0 | 0 | + |
| BT9 | F | Rectum | M | C | Through wall | No | 1/9 | 0 | 0 | + |
| BT10 | F | Sigmoid | M | C | Through wall | No | 4/5 | 0 | 0 | + |
| BT11 | M | Sigmoid | M | B | Through wall | No | 0/6 | 0 | 1 | – |
| BT12 | M | Sigmoid | W | B | Through wall | No | 0/15 | 0 | 0 | + |
| BT13 | M | Right | P | C | Through wall | No | 3/8 | 0 | 0 | – *d |
| BT14 | M | Sigmoid | M | B | Through wall | No | 0 | 0 | 2 | + |
| BT15 | M | Sigmoid | M | C | Muscularis propria | No | 1/4 | + | 0 | – |
| BT16 | M | Rectum | W | A | Muscularis propria | No | 0 | 0 | 3+ | + |
| BT17 | M | Rectum | P | C | Through wall | Yes | 9/9 | 0 | 1 | ± *d |
| BT18 | M | Right | M | B | Through wall | No | 0/7 | 0 | 0 | – |
| BT19} | M | Polyp | – | – | – | – | – | – | – | + |
| BT19} | M | Rectum | M | B | Through wall | No | 0/10 | 0 | 1 | + |
| BT20 | F | Caecum | M | C | Through wall | No | 1/8 | 0 | 0 | – |
| BT21 | F | Rectum | M | C | Through wall | No | 1 | 0 | 0 | – |
| BT22 | M | Colon | P | B | Through wall | No | 0/9 | 0 | 0 | ± |
| BT23 | F | Rectum | M | B | Through wall | No | 0/10 | 0 | 3 | + |
| BT24 | F | Right | W | B | Through wall | No | 0/7 | 0 | 0 | + |
| BT25 | F | Sigmoid | M | A | Muscularis propria | No | 0/12 | 0 | 0 | + |
| BT26 | M | Right | M | B | Through wall | No | 0/9 | 0 | 0 | + |
| BT27 | F | Rectum | M | B | Through wall | No | 0/6 | 0 | 3+ | + |
| BT28 | M | Rectum | M | B | Through wall | Yes | 0 | 0 | 0 | +** |
| BT29 | M | Sigmoid | M | B | Through wall | No | 0 | 0 | 2 | + |
| BT30 | F | Sigmoid | M | B | Through wall | No | 0/17 | + | 0 | ± |
| BT31} | M | Polyp | – | – | – | – | – | – | – | + |
| BT31} | M | Rectum | M | A | Muscularis propria | No | 0/9 | 0 | 4 | + |
| BT32} | F | Polyp | – | – | – | – | – | – | – | + |
| BT32} | F | Rectum | M | B | Through wall | No | 0/3 | 0 | 29 | + |
| BT33 | M | Polyp | – | – | – | – | – | – | – | + |
| BT34 | M | Sigmoid | M | A | Submucosa | No | 0 | 0 | 0 | – |
| BT35 | F | Sigmoid | M | C | Through wall | No | 4/7 | 0 | 0 | –* |
| BT36 | F | Sigmoid | M | B | Through wall | Yes | 0/8 | 0 | 0 | Σ** |
| BT37 | F | Right | W | A | Submucosa | No | 0/5 | 0 | 2 | – |
| BT38 | F | Rectum | M | B | Through wall | No | 0/2 | 0 | 0 | – |
| BT39 | M | Liver Met. | M | – | – | – | – | + | – | – |
| BT40} | M | Rectum | M | C | Through wall | No | 4/4 | + | 5 | ± |
| BT40} | M | Liver Met. | M | – | – | – | | + | | ± |
| BT41 | F | Rectum | P | C | Through wall | Yes | 7/10 | 0 | 0 | – |
| BT41 | M | Right | M | B | Through wall | Yes | 0/1 | 0 | 0 | +** |
| BT43 | F | Right | W | B | Through wall | No | 0/10 | 0 | 0 | – |
| BT44 | M | Sigmoid | M | B | Through wall | No | 0 | + | 3 | + |
| BT44 | M | Liver Met. | M | – | – | | – | – | – | + |

+, W, M and P denote well, moderately and poorly differentiated respectively; *Denotes recurrent disease; *d Denotes died from recurrent disease; **Denotes metastastic spread to sites other than the lymph nodes and liver.

tumour spread in large bowel tumours, shows only nine of the carcinomas from the 12 Dukes C patients, with tumour spread to the lymph nodes only, to show reduced E-cadherin expression (Table IV). Only seve of the 22 Dukes' B patients and two of the six Dukes' A patients showed reduced E-cadherin expression. Of the 42 patients studied with malignant disease, to date only three have died from recurrent disease (Table I, patients 3, 13 and 17) and only one (patient 35) has a recurrence. The carcinoma from patient 3 was well differentiated and showed high levels of E-cadherin expression, whilst the carcinomas from patients 13 and 35 were

**Table II** E-cadherin expression in the 12 primary carcinoma patients with lymph node involvement at time of surgery

| *E-cadherin expression* | *No. patients* |
|---|---|
| + | 3 |
| ± | 2 |
| – | 7 |
| Proportion with reduced E-cadherin expression | 9/12 |

**Table III** E-cadherin expression relative to differentiation status in samples from the 40 primary carcinomas

| *E-cadherin expression* | *Differentiation** *W* | *M* | *P* |
|---|---|---|---|
| + | 4 | 18 | 0 |
| ± | 0 | 3 | 2 |
| – | 2 | 9 | 2 |
| Proportion with reduced E-cadherin expression | 2/6 | 12/30 | 4/4 |

*W, M and P denot well, moderately and poorly differentiated respectively. Where corresponding liver metastases were available they had the same differentiation status as the carcinoma and exhibited identical patterns of expression.

**Table IV** E-cadherin expression relative to Dukes' stage in the primary carcinoma samples from 40 patients

| | *Dukes' stage* | | |
|---|---|---|---|
| *E-cadherin expression* | *A* | *B* | *C* |
| + | 4 | 15 | 3 |
| ± | 0 | 3 | 2 |
| – | 2 | 4 | 7 |
| Proportion with reduced E-cadherin expression | 2/6 | 7/22 | 9/12 |

poorly and moderately differentiated respectively, already had lymph node involvement and showed no E-cadherin expression. Three patients (Table I, patients 28, 36 and 42) had metastatic spread to sites other than the liver or lymph nodes. The primary carcinomas for patients 28 and 42 expressed E-cadherin whilst that of patient 36 showed reduced expression. There was no correlation between E-cadherin expression and local invasion into and through the bowel wall or with vascular invasion.

### Discussion

The aim of the present study was to evaluate the usefulness of reduced E-cadherin expression as a marker or predictor of large bowel tumour cell invasion or metastasis. However, the data summarised in Table II demonstrate that the primary carcinomas of only nine of the 12 patients with lymph node metastases show reduced E-cadherin expression. The primary carcinomas from only three of the four patients with liver involvement show reduced expression. Overall only 11 of the 17 patients with tumour spread at the time of surgery, (Table I) exhibited reduced E-cadherin expression. There was no absolute correlation for reduced E-cadherin expression with differentiation status (Table III) or Dukes' stage (Table IV) the classic prognostic indicators. The difference between the proportion of Dukes' C patients with reduced staining *vs* the proportion for Dukes' A and B patients (Table IV) was 43% with the 95% confidence interval ranging from 13 to 72%. One could interpret the fact that some of the carcinomas with lymph node involvement (Table II) and tumour spread to other sites, showed high E-cadherin expression because the assay wasn't sensitive enough to pick up the few negative staining cells that might result in metastatic foci. Alternatively, the tumour sample selected for study might not have contained negatively staining (metastatic cells) due to tumour heterogeneity or the E-cadherin although expressed might not have been functional (Hirano *et al.*, 1992).

However, the large number of carcinomas that exhibit no or reduced E-cadherin expression that have no evidence of tumour spread (Table IV) belies this. Moreover, the strong E-cadherin expression in the liver metastasis Figure 1c of the primary carcinoma of patient 44, Figure 1b, would also tend to disagree with this interpretation. Certainly the observation that the pattern of E-cadherin expression was different in the three patients (Table I, 28, 26 and 42) with metastatic spread and in the three patients (Table I, 3, 13 and 17) that have succumbed to recurrent disease, suggests that irrespective of whether or not reduced E-cadherin expression correlates with metastatic disease the routine immunofluroescent staining of tissue sections is not precise enough to be used as part of routine pathology.

Overall, one has to conclude that reduced E-cadherin expression is observed more frequently with tumour spread, but in isolation reduced E-cadherin expression is probably not a predictor of metastatic potential in large bowel tumours. A significant proportion of the tumours that stain negatively and therefore would if the hypothesis was accurate contain large numbers of invasive or metastatic cells, have no evidence of metastatic spread or rapid recurrent disease at the present time. Obviously long-term the outcome and disease free intervals for all the patients need to be considered. Patient 1 in the series was only operated on 18 months ago and more meaningful results will be obtained once follow up gets out to 5 years. However, these preliminary observations would seem to support the observations of Shimoyama and co-workers that there is no simple relationship between E-cadherin expression and increased invasiveness for all human carcinomas (Shimoyama *et al.*, 1989; Shimoyama & Hirohashi, 1991*b*). Tumour cell invasion and metastasis is known to be a complex process and it may be that other critical gene products such as nm23 (Steeg *et al.*, 1991), CD44 (Anstee *et al.*, 1991) and components of the cytoskeleton have a critical role to play in the process in certain tumour types.

The authors would like to thank Professor R. Shields, Mr R. Sutton, Mr G. Poston and Mr J. Winstanley for help with the provision of tumour material, Mr P. O'Brien for preparing the frozen sections and Professor W. Birchmeier, University of Essen, Germany, for the 6F9 antibody, advice and encouragement. The work was funded by a NWCRF grant to Dr Anne Kinsella.

### References

ANSTEE, D.J., GARDNER, B., SPRING, F.A., HOLMES, C.H., SIMPSON, K.L., PARSONS, S.F., MALLINSON, G., YOUSAF, S.M. & JUDSON, P.A. (1991). New monoclonal antibodies to CD44 and CD58: their use to quantify CD44 and CD58 on normal human erythrocytes and to compare the distribution of CD44 and CD58 in human tissues. *Immunology*, **74**, 197–205.

ASTLER, V.B. & COLLER, F.A. (1954). The prognostic significance of direct extentions of carcinoma of the colon and rectum. *Ann. Surg.*, **139**, 846.

BEHRENS, J., MAREEL, M.M., VAN ROY, F.M. & BIRCHMEIER, W. (1989). Dissecting tumour cell invasion: epithelial cells acquire invasive properties after the loss of uvomorulin-mediated cell-cell adhesion. *J. Cell Biol.*, **108**, 2435–2447.

DUKES, C.E. (1936). Histological grading of rectal cancer. *Proc. R. Soc. Med.*, **30**, 371–376.

EIDELMAN, G.M., GALLIN, W.J., DLOUVEE, A., CUNNINGHAM, B.A. & THIERY, J.-P. (1989). Expression of the cell-cell adhesion glycoprotein cell-CAM 120/80 in normal human tissue and tumours. *Am. J. Pathol.*, **135**, 101–110.

FRIXEN, U.H., BEHRENS, J., SACHS, M., EBERLE, G., VOSS, B., WARDA, A., LOCHNER, D. & BIRCHMEIER, W. (1991). E-cadherin mediated cell-cell adhesion prevents invasiveness of human carcinoma cells. *J. Cell Biol.*, **113**, 175–185.

HASHIMOTO, M., NIWA, O., NITTA, Y., TAKEICHI, M. & YOKORO, K. (1989). Unstable expression of E-cadherin adhesion molecules in metastatic ovarian tumour cells. *Jpn. J. Cancer Res.*, **80**, 459–463.

HIRANO, S., KIMOTO, N., SHIMOYAMA, Y., HIROHASHI, S. & TAKEICHI, M. (1992). Identification of a neural -catenin as a key regulator of cadherin function and multicellular organisation. *Cell*, **70**, 293–301.

MEGE, R.-M., MATSUZAKI, F., GALLIN, W.J., GOLDBERG, J.L., CUNNINGHAM, B.A., EDELMAN, G.M. (1988). Construction of epithelioid sheets by transfection of mouse carcinoma cells with cDNAs for chicken cell adhesion molecules. *Proc. Natl Acad. Sci. USA*, **85**, 7274–7278.

NAGAFUCHI, A., SHIRAYOSHI, Y., OKAZAKI, K., YASUDA, K. & TAKEICHI, M. (1987). Transformation of cell adhesion properties by exogenously introduced E-cadherin cDNA. *Nature*, **329**, 341–343.

PFISTERER, J., WINTZER, O., FRIXEN, U.H., BEHRENS, J., EBERLE, G., BIRCHMEIER, W. & BAUKNECHT, T. (1990). Arc-1/uvomorulin by immunochemistry in non-malignant tissue and in ovarian tumours. *J. Cancer Res. Clin. Oncol.*, **116**, 182.

SCHIPPER, J., FRIXEN, U.H., BEHRENS, J., UNGER, A., JAHNKE, K. & BIRCHMEIER, W. (1991). E-cadherin expression in squamous cell carcinomas of the head and neck: invasive correlation with tumour dedifferentiation and lymph node metastasis. *Cancer Res.*, **51**, 6328-6337.

SHIMOYAMA, Y. & HIROHASHI, S. (1991*a*). Cadherin intercellular adhesion molecule in hepatocellular carcinoma: loss of E-cadherin expression in an undifferentiated carcinoma. *Cancer Letts*, **37**, 131-135.

SHIMOYAMA, Y. & HIROHASHI, S. (1991*b*). Expression of E- and P-cadherin in gastric carcinomas. *Cancer Res.*, **51**, 2185-2192.

SHIMOYAMA, Y., HIROHASHI, S., HIRANO, S., HOGUCHI, M., SHIMOSATO, Y., TAKEICHI, M. & ABE, O. (1989). Cadherin cell adhesion molecules in human epithelial tissue and carcinomas. *Cancer Res.*, **49**, 2128-2133.

STEEG, P.S., COHN, K.H. & LEONE, A. (1991). Tumour metastasis and nm23: current concepts. *Cancer Cells*, **3** (7), 257-262.

TAKEICHI, M. (1988). The cadherins: cell-cell adhesion molecules controlling animal morphogenesis. *Development*, **102**, 639-655.